# Persistent retinal tenting in myopic traction maculopathy

**DOI:** 10.1016/j.ajoc.2026.102618

**Published:** 2026-06-11

**Authors:** Taku Wakabayashi, Bita Momenaei, Allen Chiang

**Affiliations:** Wills Eye Hospital, Mid Atlantic Retina, Thomas Jefferson University, Philadelphia, PA, USA

## 1 Case report

**Fig. 1:**
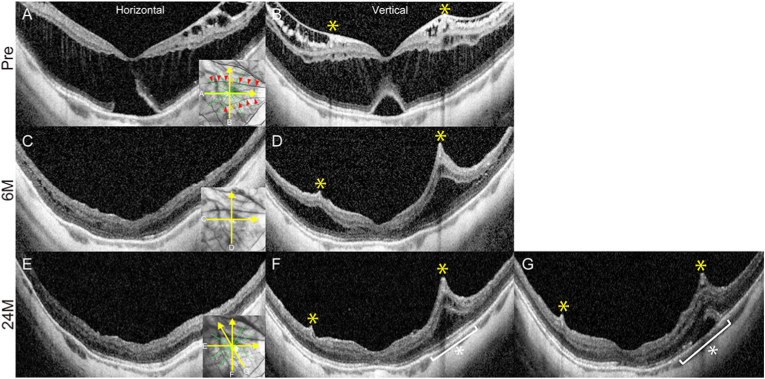
Optical coherence tomography (OCT) image before and after pars plana vitrectomy, internal limiting membrane (ILM) peeling, and 20% SF6 gas tamponade for myopic traction maculopathy (MTM) with foveal detachment in a 64-year-old woman. (**A**) Horizontal OCT image. (**B**) Vertical OCT image. OCT images before surgery showed MTM with foveal detachment resulting from traction exerted by epiretinal membrane, ILM, and retinal vessels (red arrowheads in **A**, yellow asterisks in **B**) traversing near the macula. Her visual acuity was 20/60 OD. (**C**) Horizontal OCT at 6 months after surgery showed schisis resolution. (**D**) Vertical OCT at 6 months showed persistent retinal tenting and schisis (yellow asterisks). (**E**) Horizontal OCT at 24 months after surgery showed schisis resolution. (**F**) Vertical OCT shows persistent unresolved retinal tenting and schisis (yellow asterisks). (**G**) Focal retinal detachment (white asterisks) and persistent schisis (yellow asterisks) were observed associated with retinal tenting. However, her visual acuity improved to 20/20 OD because persistent schisis and detachment did not extend toward the fovea. (For interpretation of the references to colour in this figure legend, the reader is referred to the Web version of this article.)

A 64-year-old woman presented with myopic traction maculopathy, with traction exerted by epiretinal membrane (ERM), internal limiting membrane (ILM), and retinal vessels (red arrowheads in **A**, yellow asterisks in **B**) traversing near the macula. Her visual acuity was 20/60 OD. After pars plana vitrectomy, ILM peeling from arcade to arcade, and 20% sulfur hexafluoride (SF6) gas tamponade, her visual acuity improved to 20/20 OD over 24 months. Postoperative optical coherence tomography (OCT) showed gradual resolution of schisis horizontally, but persistent retinal tenting, schisis, and focal detachment vertically associated with retinal arterioles (yellow and white asterisks) (**C-G**) (Fig. 1).

## 2 Discussion

Myopic traction maculopathy is characterized by splitting of the neurosensory retina, resulting from tangential traction from the vitreous cortex remnants and epiretinal membranes, anteroposterior traction associated with posterior staphyloma, and increased rigidity of the inner retina associated with vitreous cortex remnants, ERMs, ILM, and retinal vessels. Inner retinal elevations along retinal vessels, originally described as retinal vascular microfolds, are often seen on vertical OCT and represent vessel-induced traction on the retinal surface in MTM.^1^ During vitrectomy, vitreous cortex remnants, ERMs, and ILM can be successfully peeled; however, traction exerted by retinal vessels cannot be surgically removed. Consequently, traction exerted by retinal vessels especially artery and arterioles may persist, resulting in residual inward forces on the inner retina and potential recurrence of schisis and localized retinal detachment.^2^ We used the term “retinal tenting” in the title because the persistent vascular traction produced a tent-shaped retinal elevation on OCT. Although this finding represents the same pathology as the retinal microfolds originally described by Ikuno et al.,^2^ the traction and retinal elevation appeared to be more pronounced in our case, resulting in a more tent-like configuration. Although complete schisis resolution may be achieved at the fovea after surgery, retinal vessels-induced traction may persist in eyes with posterior staphyloma, leading to localized retinal tenting and incomplete resolution of schisis or detachment. According to a previous report by Ikuno et al., retinal vascular microfolds were observed in 62% of eyes after vitrectomy for myopic foveoschisis. However, localized retinal detachment was not mentioned in their study. In our case, localized retinal detachment and incomplete resolution of the schisis were observed even 24 months after vitrectomy in association with persistent traction caused by the retinal vessels. Retinal vessels that caused retinal tenting were arterioles in our case; however, the degree of retinal tenting and schisis resolution differed between superior and inferior retinal arterioles. The superior arteriole, in which retinal tenting was more prominent, had a larger diameter compared to inferior arteriole, potentially resulting in greater vascular traction and less resolution of the schisis. The current patient experienced good visual recovery as the persistent schisis and detachment did not extend toward the fovea. However, in eyes with retinal vessels traversing close to the fovea, persistent or recurrent schisis and focal retinal detachment associated with retinal tenting may adversely affect visual outcomes, and this possibility should be considered during postoperative follow-up.

## 3 Conclusion

In eyes with MTM, complete schisis resolution may not always be achieved due to persistent vascular traction near the macula despite successful vitrectomy and ILM removal.

## CRediT authorship contribution statement

**Taku Wakabayashi:** Writing – original draft, Data curation, Conceptualization. **Bita Momenaei:** Writing – review & editing, Validation. **Allen Chiang:** Writing – review & editing, Validation, Supervision.

## Patient consent

Written consent to publish this case has not been obtained. This report does not contain any personal identifying information.

## Authorship

All authors attest that they meet the current ICMJE criteria for Authorship.

## Artificial Intelligence statement

Artificial Intelligence was not used in preparation of the manuscript.

## Financial support

The authors received no financial support for the research of this article.

## Declaration of competing interest

The authors declare that they have no known competing financial interests or personal relationships that could have appeared to influence the work reported in this paper.
